# The Neandertal Progesterone Receptor

**DOI:** 10.1093/molbev/msaa119

**Published:** 2020-05-21

**Authors:** Hugo Zeberg, Janet Kelso, Svante Pääbo

**Affiliations:** m1 Max Planck Institute for Evolutionary Anthropology, Leipzig, Germany; m2 Department of Neuroscience, Karolinska Institutet, Stockholm, Sweden; m3 Okinawa Institute of Science and Technology, Onna-son, Japan

**Keywords:** progesterone receptor, Neandertals, fertility, PROGINS

## Abstract

The hormone progesterone is important for preparing the uterine lining for egg implantation and for maintaining the early stages of pregnancy. The gene encoding the progesterone receptor (*PGR*) carries introgressed Neandertal haplotypes with two missense substitutions and a mobile *Alu* element. These Neandertal gene variants have reached nearly 20% frequency in non-Africans and have been associated with preterm birth. Here, we show that one of the missense substitutions appears fixed in Neandertals, while the other substitution as well as the *Alu* insertion were polymorphic among Neandertals. We show that two Neandertal haplotypes carrying the *PGR* gene entered the modern human population and that present-day carriers of the Neandertal haplotypes express higher levels of the receptor. In a cohort of present-day Britons, these carriers have more siblings, fewer miscarriages, and less bleeding during early pregnancy suggesting that the Neandertal progesterone receptor alleles promote fertility. This may explain their high frequency in modern human populations.

Progesterone is a steroid sex hormone produced by the ovaries, placenta, and adrenal glands that is involved in pregnancy, menstrual cycle, libido, and embryogenesis in placental mammals (Taraborrelli 2015). The progesterone receptor is encoded by the *PGR* gene on chromosome 11, and is most highly expressed in the endometrium. Binding of progesterone (or synthetic progestins) to the receptor mediates a gene regulation cascade that converts the endometrium to its secretory stage to prepare the uterus for implantation and helps maintain pregnancy. Progesterone is also involved in stimulation of the mammary glands during pregnancy. It also has less well-understood roles as a neurosteroid in the brain (Baulieu and Schumacher 2000).

A polymorphic variant of *PGR* which carries the missense substitution V660L (rs1042838) in exon 4 and an *Alu* insertion between exons 7 and 8 occurs among present-day populations, reaching frequencies of up to ∼20% (sometimes called “PROGINS”; Rowe et al. 1995; Agoulnik et al. 2004; Terry et al. 2005; Liu et al. 2014). A haplotype containing the V660L substitution and the *Alu* insertion as well as a synonymous mutation (H770H, rs1042839, exon 5) has been associated with preterm birth (Tiwari et al. 2015; Li et al. 2018), ovarian and endometrial cancer (Liu et al. 2014), migraine (Palmirotta et al. 2015), and endometriosis (Wieser et al. 2002). Two functional studies have come to conflicting conclusions with respect to the responsiveness of variants of the receptor and their stability (Romano et al. 2007; Stenzig et al. 2010), perhaps depending on the particular variants and cell types used.

It has recently been noted (Li et al. 2018) that the valine to leucine substitution at position 660 occurs in a homozygous form in two Neandertal genomes sequenced to high coverage whereas it is not present in the genome of a Denisovan, an Asian relative of the Neandertals. We find that it is also homozygously present in a third Neandertal genome and present in Neandertal genomes sequenced to low coverage (supplementary table S2, Supplementary Material online). The association with preterm birth has been taken to indicate that it conferred an evolutionary disadvantage on Neandertals (Li et al. 2018) and raises the question why it has risen in frequency in modern human populations. Here, we revisit the Neandertal progesterone receptor in the light of recent data.

The V660L variant occurs at frequencies between 2% and 22% among Europeans and Native Americans as well as in parts of Asia (fig. 1*A*). It sits on a DNA segment of at least 56 kb (*r*^2^ > 0.8) that is introgressed from Neandertals (fig. 1*B*, *P* = 0.02, Materials and Methods). In addition to V660L, the Neandertal haplotype includes the H770H synonymous variant (rs1042839, *r*^2^ = 0.98) and the S344T missense variant (rs3740753, *r*^2^ = 0.95), both of which were polymorphic in Neandertals but do not occur in the high coverage Denisovan genome (supplementary table S2, Supplementary Material online). The *Alu* element is embedded in the Neandertal haplotype but we find that V660L does not fully cosegregate with *Alu* insertion in the 1000 Genomes data set (*r*^2^ = 0.72).

**Fig. 1:**
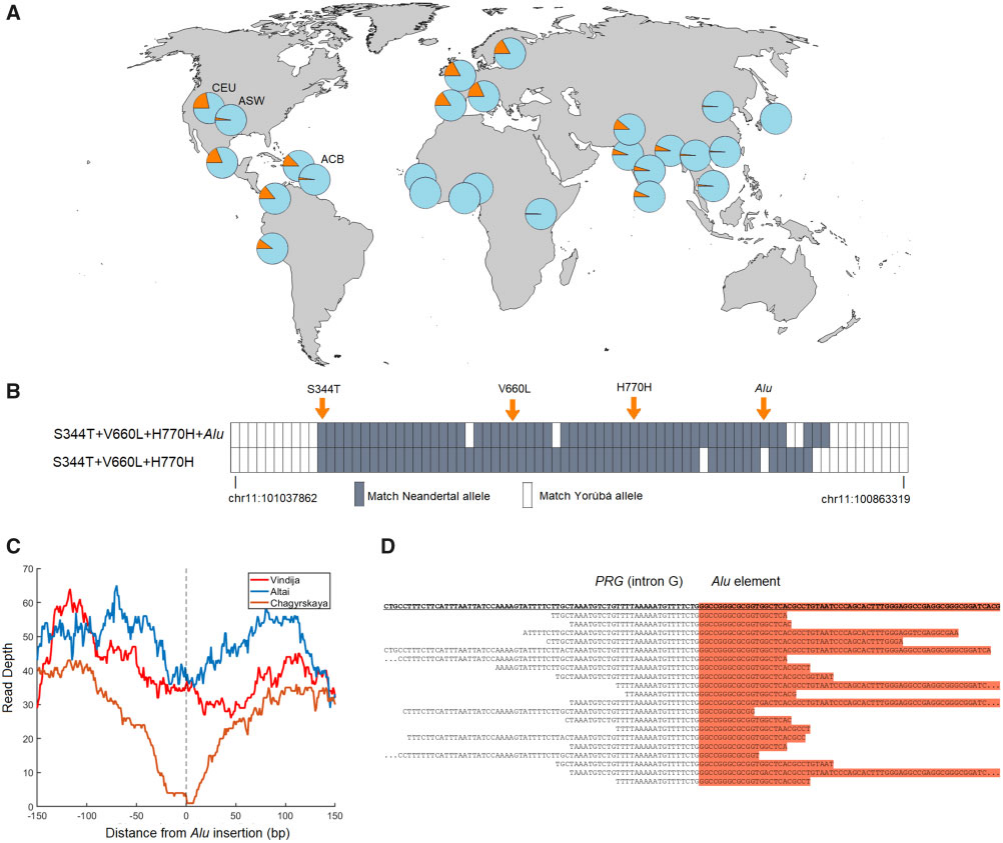
Geographic distribution of V660L, introgressed Neandertal haplotypes and the *Alu* element in *PGR*. (*A*) Allele frequency of V660L (rs1042838) in 26 populations. Data from the phase 3 release of the 1000 Genomes Project. The African American populations (ASW, African Ancestry in Southwest United States; ACB, African Caribbeans in Barbados) have a lower frequency of V660L, similar to that in African populations, whereas North Americans with European ancestry (e.g., CEU: Utah Residents with Northern and Western European Ancestry) have frequency similar to European populations. (*B*) Similarity of present-day chromosomes carrying S344T+V660L+H770H with (*n* = 256) and without (*n* = 89) the *Alu* element to three high-coverage Neandertal genomes. In addition to the four polymorphisms indicated, data for 74 SNPs where a minor or absent allele among the Yoruba in the 1000 Genomes Project occurs three or more times among the three Neandertals. A gray box indicates that the major allele among the chromosomes with (above) or without (below) the *Alu* insertion matches the allele that occurs three or more times among the Neandertals. (*C*) Read depth for three high-coverage Neandertal genomes when aligned to the reference human genome (hg19). Note the symmetrical drop in read depth at the site for the *Alu* insertion in the Chagyrskaya Neandertal genome and a less pronounced drop in coverage in the Altai genome. (*D*) DNA fragments from the Chagyrskaya genome aligned to a DNA sequence carrying the *Alu* element around the 5′-end of the *Alu* element (chr11:100,911,793).

The Neandertal haplotypes with and without the *Alu* element exist in all major populations of present-day non-Africans (fig. 1*A*;supplementary table S1, Supplementary Material online). This suggests that the *Alu* insertion took place early after introgression of the haplotype into modern humans or that it was polymorphic among Neandertals and that at least two Neandertal haplotypes were transferred to modern humans. To determine whether the *Alu* insertion was polymorphic among Neandertals we analyzed shotgun sequence data of the three high-coverage Neandertal genomes flanking the site of the *Alu* insertion. If the insertion was homozygously absent in a Neandertal we expect the coverage to match the genomic average around the insertion site, as the reference human genome (*hg19*) to which the Neandertal sequences are aligned does not carry the *Alu* insertion. In contrast, if the *Alu* element is homozygously present we expect the read depth to drop to near zero as short ancient DNA fragments fail to align. For one 60–80,000-year-old Neandertal genome from Siberia, coverage drops symmetrically to zero around the site of the *Alu* insertion (fig. 1*C*) and no fragments covering the *Alu* insertion site are seen, suggesting that the *Alu* element was homozygously present in this Neandertal. We aligned the DNA fragments sequenced from this genome to the haplotype carrying the *Alu* insertion and found 20 fragments carrying the 5′-end of the *Alu* element as well as adjacent single-copy DNA sequences (fig. 1*D*, Materials and Methods). In another ∼120,000-year-old Siberian Neandertal genome, a less pronounced reduction of the coverage was observed at the site of the *Alu* element. For that genome, we found 25 fragments carrying the *Alu* element and adjacent single-copy sequences and 40 fragments covering the *Alu* insertion site without the insertion, suggesting that this Neandertal was heterozygous for the *Alu* insertion. In contrast, 36 and 0 fragments without and with the *Alu* insertion, respectively, were found in an ∼50,000-year-old Neandertal genome from Europe, suggesting that this individual homozygously lacked the insertion. Thus, two variants of the Neandertal *PGR* haplotype existed among Neandertals, one with and one without the *Alu* element, and both were introduced into the gene pool of modern humans.

As the number of sequenced ancient modern human genomes increases, it is becoming possible to follow changes in the frequency of genetic variants over time in modern humans. The oldest modern human individual available who carries the Neandertal-derived V660L variant is an ∼40,000-year-old individual from Tianyuan Cave, China (Yang et al. 2017). The V660L variant is then present in several Pleistocene genomes west of the Ural Mountains older than 10,000 years (fig. 2*A*; Reich 2019; see also Supplementary Movie) and becomes progressively more common in western Eurasia after that time (fig. 2*B*). When more ancient genomes become available from East Asia, it will hopefully be possible to address why a corresponding increase is not seen there (fig. 1*A*).

**Fig. 2:**
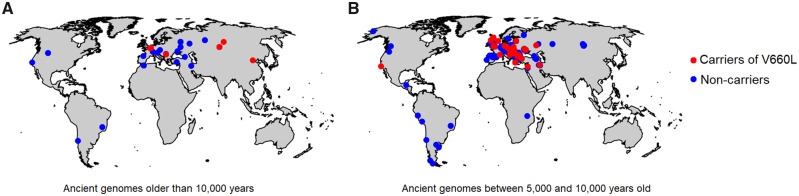
Geographic distribution of ancient genomes carrying the Neandertal-derived V660L variant. (*A*) Ancient genomes older than 10,000 years. (*B*) Ancient genomes between 5,000 and 10,000 years old.

To determine whether the Neandertal haplotype influences phenotypic traits in modern carriers we searched for associations between V660L polymorphism and phenotypes among 452,264 Britons in the UK Biobank using the Gene ATLAS tool (Canela-Xandri et al. 2018). Of 22 inpatient diagnoses related to pregnancy, childbirth and the puerperium (chapter XV of the International Classification of Diseases; ICD), we find a negative association between the Neandertal allele and “hemorrhage in early pregnancy” (ICD O20; OR = 0.83, *P* = 0.002, *P*(adjusted) = 0.044; fig. 3*A*). In the UK Biobank interview records, carriers of the Neandertal allele report less miscarriages (OR = 0.85, nominal significant at *P* = 0.009, although not when corrected for multiple testing; fig. 3*A*). As a proxy for fertility, we use the number of full sisters and brothers, although only half of the individuals carrying one copy of the Neandertal allele would have a mother with the Neandertal variant. Nevertheless, individuals carrying the Neandertal V660L allele have significantly more sisters (*P* = 0.0036; fig. 3*B*) than those carrying the ancestral allele, whereas there is no difference for brothers. Taken together, the increased number of sisters and the reduced risk of bleeding and miscarriages suggest that the Neandertal variant of *PGR* is associated with increased fertility.

**Fig. 3:**
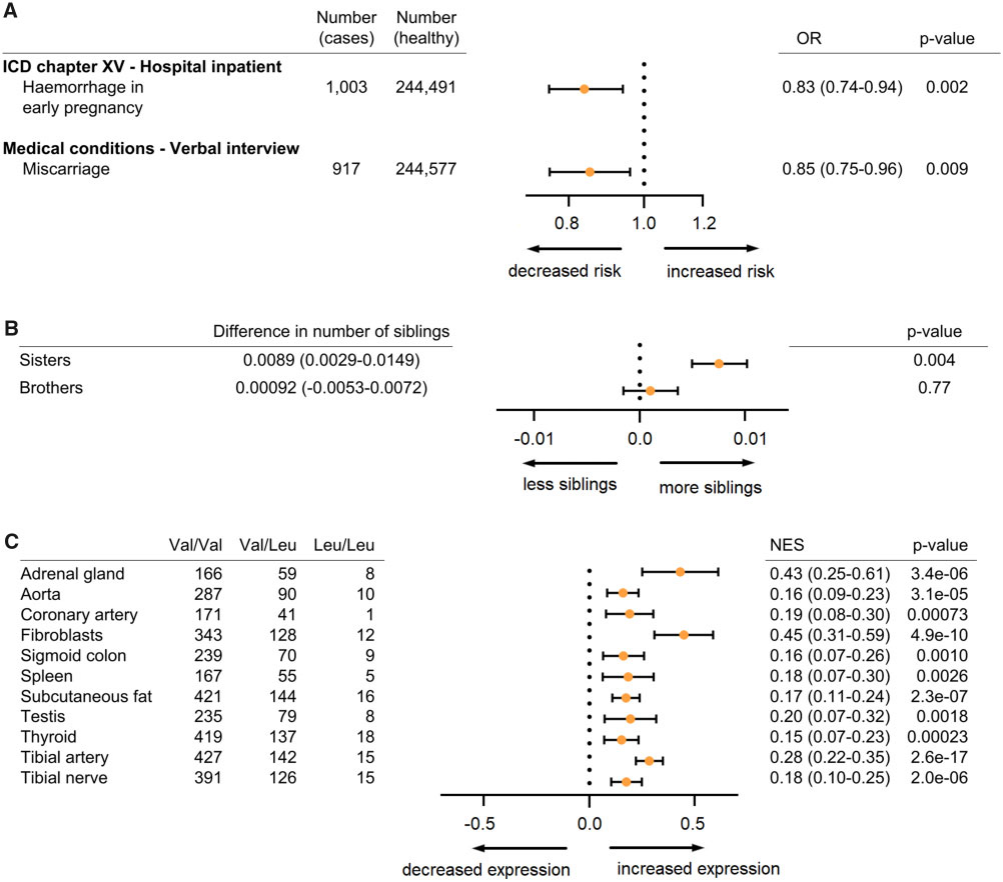
Phenotypic consequences and relative expression levels associated with the V660L allele. (*A*) Odds ratios (OR) for hemorrhage in early pregnancy (ICD O20) and miscarriage for carriers of the V660L allele. (*B*) Numbers of full sisters and full brothers for the V660L allele. 95% confidence intervals in parentheses. Frequency of the V660 allele in the cohort is 16.5%. (*C*) Normalized effect sizes (NES) for 11 tissues where V660L is predicted to explain variation in expression levels of *PGR* mRNA (posterior probability of effect > 0.9). In all 11 tissues, the Neandertal allele is associated with higher expression; 95% confidence intervals in parentheses.

To investigate if the V660L polymorphism affects the expression of the progesterone receptor, we use data from the Genotype-Tissue Expression project (GTEx). The eleven tissues with a posterior probability of an effect >0.9 (Han and Eskin 2012) are shown in figure 3*C*. We find that V660L is associated with higher mRNA expression of the progesterone receptor (*P*=–7.00e-51; meta-analysis across all tissues).

Orally administered progesterone has been shown to reduce the rate of spontaneous miscarriages and to improve fertility among women who have experienced bleeding in early pregnancy and recurrent miscarriages (Haas et al. 2019). Given the role of progesterone in the maintenance of pregnancy (Taraborrelli 2015), some effect of the Neandertal *PGR* haplotypes, particularly their higher expression (fig. 3*C*), may explain their association with increased fertility and why they appear to have increased in frequency over time in Europe and the Americas (figs. 1 and 2). This increase in frequency is in apparent contradiction to their association with preterm births (Tiwari et al 2015; Li et al 2018). However, we suggest that the Neandertal progesterone receptor variants may help maintain pregnancies that would otherwise be terminated, and that a consequence (or physiological trade-off) of this may be the association of the same variants with preterm live births. There also seems to be no grounds to assume that the Neandertal versions of *PGR* posed a selective disadvantage to Neandertals. In fact, the association with higher numbers of live births might explain why some of these derived changes seem to have become frequent or fixed among Neandertals, although the smaller effective population size of Neandertals might have reduced the effectiveness of selection in Neandertals (Castellano et al 2014; Harris and Nielsen, 2016).

## Materials and Methods

### Evidence for Introgression

The V660L polymorphism sits on a 56.2-kb-long haplotype (*r*^2^>0.8 in all 1000G individuals) defined by 28 private SNPs on the Neandertal lineage (i.e., the Neandertal allele is missing in 108 Yoruba individuals) with coordinates chr11:100877202–100933412 (hg19). Inserting this length into the formula derived by Huerta-Sánchez et al. (2018) yields a probability of *P* = 0.02 for incomplete ancestral lineage sorting (ILS), using a generation time of 25 years, the local recombination rate of 0.87 cM/Mb (deCODE; Kong et al. 2010), and a branch length of 200 ky for the modern human branch, and 100 ka for the Neandertal branch. We use a conservative lower estimate of the branch lengths because the formula underestimates the probability of ILS if the branch lengths are overestimated. Previous genome-wide analyses (Sankararaman et al. 2014; Danneman and Kelso 2017; Li et al. 2018) have similarly indicated this locus as carrying introgressed Neandertal haplotypes.

### Detecting Mobile Elements in Archaic Genomes

If an insertion is not present in the reference genome, the read depth should drop (to zero if homozygous) in a symmetrical fashion around the position of insertion. This pattern is observed for the Chagyrskaya Neandertal genome. A smaller drop is seen for the Altai Neandertal genome. We realigned the sequenced fragments to a reference sequence containing the *Alu* insertion. A sequence 11 bp upstream to the *Alu* insertion and 11 bp into the *Alu* insertion (that is unique in the entire NCBI database) was used to identify junction fragments.

### A Neandertal *PGR* without the *Alu* Element

We find that V660L is not in particularly high linkage disequilibrium with the *Alu* insertion (*r*^2^ = 0.72). To rule out sequencing error, we verified this in two of the 24 high-coverage 1000G genomes, which carried V660L but not the *Alu* insertion (NA19625 and HG01500). Linkage disequilibrium between V660L and the *Alu* element for all 1000G subpopulations is given supplementary table S1, Supplementary Material online.

### Phenotypic Consequences

We investigated phenotypic associations in the UK Biobank using the GeneAtlas tool (Canela-Xandri et al. 2018). In brief, the Gene ATLAS provides 778 associations based on Mixed Linear Models using 452,264 Britons with European descent. As fixed effects, the model includes sex, array batch, UK Biobank Assessment Center, age, and 20 genomic principal components. Population structure was captured as a random effect. Both V660L (rs1042838) and S344T (rs3740753) are included in UK Biobank SNP array, so no imputation was needed for these. However, the *Alu* insertion is not genotyped and our analyses are thus limited to the missense variants of the haplotype. *P*-values were adjusted for multiple comparisons by controlling for the family-wise error rate (supplementary table S3, Supplementary Material online). Confidence intervals for the odds ratios were calculated using the method described in Altman and Bland (2011). Of the 778 association in the GeneAtlas, 22 where classified as belonging to ICD chapter XV (Pregnancy, childbirth, and the puerperium, supplementary table S3, Supplementary Material online). In addition, we identified 53 traits, which we considered to be related to the female reproductive system. For V660L we found negative correlations with “hemorrhage in early pregnancy” (*P* = 0.002), miscarriage (*P* = 0.01), and positive correlation with more sisters (*P* = 0.0036). This result was replicated for S344T (*P* = 0.0006, *P* = 0.028, and *P* = 0.00097, respectively). *P*-values and odds ratios for V660L are shown in figure 3 and supplementary table S3, Supplementary Material online.

We analyzed the age of menarche and menopause using the Biobank Japan (Horikoshi et al. 2018). Among 43,861 Japanese women, the Neandertal haplotype is associated with a somewhat earlier menopause (rs585447, 0.19 years per allele, *P* = 0.048) whereas there is no effect on menarche. *P*-values and effect sizes were taken directly from Biobank Japan.

We analyzed the effect of V660L (rs1042838) on PGR mRNA expression levels using the version eigth release of the Genotype-Tissues Expression (GTEx) project. Tissues which were predicted to show an effect were selected based on a posterior probability (i.e., *m*-value) >0.9. The effect across tissues was estimated using Han and Eskin’s Random Effects model (RE2; Han and Eskin 2012).

## Supplementary Material

Supplementary data are available at *Molecular Biology and Evolution* online.

## Supplementary Material

msaa119_Supplementary_Data
