# Supplementary material for: The Neandertal Progesterone Receptor
Source: Mol Biol Evol. 2020 May 21;37(9):2655–60. doi: 10.1093/molbev/msaa119 (PMC7475037; doi:10.1093/molbev/msaa119)
Supplement: msaa119_Supplementary_Data [file msaa119_supplementary_data.zip › msaa119-Suppl_Data/Supplementary_Materials_270420_clean.pdf]

	Frequency (%)				LD with the *Alu* element (r^2)		
	S344T	V660L	H770H	*Alu*	S344T	V660L	H770H
**East Asia**							
CHB	1.0	1.0	1.0	1.0	1.00	1.00	1.00
JPT	0.0	0.0	0	0.0			
CHS	1.0	1.0	1.0	1.0	1.00	1.00	1.00
CDX	1.6	1.6	1.6	1.6	1.00	1.00	1.00
KHV	1.5	1.5	1.5	1.0	0.66	0.66	0.66
**South Asia**							
GIH	5.8	5.8	5.8	4.9	0.82	0.82	0.82
PJL	11.5	10.9	10.9	8.9	0.75	0.79	0.79
BEB	7.0	5.8	5.2	3.5	0.48	0.59	0.65
STU	6.4	6.4	4.4	3.4	0.52	0.52	0.77
ITU	4.4	4.4	4.4	3.4	0.77	0.77	0.77
**Europe**							
CEU	21.2	21.7	21.2	17.2	0.71	0.75	0.71
TSI	17.8	18.2	18.7	16.8	0.65	0.85	0.88
FIN	16.7	16.7	16.7	10.1	0.56	0.56	0.56
GBR	17.0	17.0	17.0	13.2	0.74	0.74	0.74
IBS	15.9	15.9	15.9	9.3	0.55	0.55	0.55
**Africa**							
YRI	0.0	0.0	0.0	0.0			
LWK	0.5	0.5	0.5	0.0			
GWD	0.0	0.0	0.0	0.0			
MSL	0.0	0.0	0.0	0.0			
ESN	0.0	0.0	0.0	0.0			
**America**							
ASW*	2.5	2.5	2.5	0.8	0.33	0.33	0.33
ACB*	2.1	2.1	2.1	1.6	0.75	0.75	0.75
MXL	19.5	18.8	18.8	14.1	0.67	0.71	0.71
PUR	13.0	13.0	12.5	8.2	0.60	0.50	0.62
CLM	14.9	14.4	14.4	10.1	0.64	0.67	0.67
PEL	10.0	10.0	10.0	9.4	0.81	0.94	0.94

Table S1. Frequencies of the three Neandertal-derived substitutions as well as the *Alu* insertion in 1000G populations and linkage disequilibrium (LD) between the single-nucleotide polymorphisms and the *Alu* insertion. CHB, Han Chinese in Beijing; JPT, Japanese in Tokyo; CHS, Southern Han Chinese; CDX, Chinese Dai in Xishuangbanna; KHV, Kinh in Ho Chi Minh City; GIH, Gujarati Indian from Houston, Texas; PJL, Punjabi from Lahore; BEB, Bengali from Bangladesh; STU, Sri Lankan Tamil from the UK; ITU, Indian Telugu from the UK; CEU, Utah Residents with Northern and Western European Ancestry; TSI, Toscani in Italia; FIN, Finnish in Finland; GBR, British in England and Scotland; IBS Iberian Population in Spain; YRI, Yoruba in Ibadan; LWK, Luhya in Webuye; GWD, Gambian in Western Divisions; MSL, Mende in Sierra Leone; ESN, Esan in Nigeria; ASW, Americans of African Ancestry in SW USA; ACB, African Caribbeans in Barbados; MXL, Mexican Ancestry from Los Angeles; PUR, Puerto Ricans; CLM, Colombians from Medellin; PEL, Peruvians from Lima. Note that the African American populations* (ASW, ACB) have low allele frequencies of V660L, similar to African populations.

	S344T	V660L	H770H	*Alu*
**High-coverage genomes**				
Altai	0/1	1/1	0/1	0/1
Vindija	1/1	1/1	0/0	0/0
Chagyrskaya	1/1	1/1	1/1	1/1
Denisova	0/0	0/0	0/0	0/0
**Intermediate-coverage genomes**				
El Sidrón	./.	1/1	0/0	./.
**Low-coverage genomes**				
Mezmaiskaya 1	1	1	1	1
Mezmaiskaya 2	0	1	0	.
Goyet	1	1	0	.
Les Cottes	0	1	0	.
Spy	1	.	0	.
Forbes' Quarry	.	.	.	.
Hohlenstein-Stadel	.	.	.	.
Scladina	.	.	.	.
Sima de los Huesos	0	.	.	.
Denisova 11	.	1	0	.

Table S2. Neandertal-derived amino-acid substitutions and the *Alu* insertion in archaic genomes. The presence of the *Alu* insertion in the high-coverage genomes (Meyer et al. 2012, Prüfer et al. 2014, Prüfer et al. 2017) is based on coverage and reads overlapping the insertion, as described in Supplemental Material and Methods. '0' indicates a match to the human reference genome (*i.e.*, S344, V660, H770 and the absence of the *Alu* insertion), '1' indicates the non-reference allele and '.' missing data. *Denisova 11* has a Neandertal father and a Denisovan mother (Slon et al. 2018) For the three high-coverage genomes, as well as for El Sidrón, we report genotypes. The El Sidrón genome was exomes captured to a coverage of 12.5-fold (Castellano el al. 2014). For the low coverage genomes (Hajdinjak et al. 2018), one fragment covering each of the three polymorphism were randomly drawn.

Meyer, M. et al. 2012. Science 338, 222-226
Prüfer, K. et al. 2014. Nature 505, 43-49
Prüfer, K. et al. 2017. Science 358, 655-658
Slon, V. et al 2018. Nature. 561:113–116
Castellano, S. et al. 2014. Proc. Natl. Acad. Sci. USA 111:6666-6671

	OR	p-value	p-value (adjust)
**ICD chapter XV (Pregnancy, childbirth and the puerperium)**			
O02 Other abnormal products of conception	1.03	0.529	0.979
O03 Spontaneous abortion	0.99	0.907	0.979
O04 Medical abortion	0.98	0.709	0.979
O16 Unspecified maternal hypertension	1.02	0.831	0.979
O20 Haemorrhage in early pregnancy	0.83	**0.002**	**0.044**
O26 Maternal care for other conditions predominantly related to pregnancy	0.93	0.055	0.593
O32 Maternal care for known or suspected malpresentation of foetus	1.00	0.972	0.979
O34 Maternal care for known or suspected abnormality of pelvic organs	1.01	0.765	0.979
O36 Maternal care for other known or suspected foetal problems	0.99	0.772	0.979
O42 Premature rupture of membranes	1.05	0.373	0.979
O46 Antepartum haemorrhage, not elsewhere classified	0.95	0.473	0.979
O47 False labour	0.99	0.876	0.979
O48 Prolonged pregnancy	1.00	0.929	0.979
O63 Long labour	0.98	0.569	0.979
O68 Labour and delivery complicated by foetal stress [distress]	1.06	0.081	0.593
O69 Labour and delivery complicated by umbilical cord complications	1.01	0.859	0.979
O70 Perineal laceration during delivery	1.01	0.717	0.979
O72 Postpartum haemorrhage	1.07	0.192	0.979
O75 Other complications of labour and delivery, not elsewhere classified	0.94	0.383	0.979
O80 Single spontaneous delivery	0.97	0.406	0.979
O82 Single delivery by Caesarean section	1.00	0.979	0.979
O99 Other maternal diseases classifiable elsewhere but complicating pregnancy, childbirth and the puerperium	1.00	0.938	0.979

Table S3. Phenotypic associations between V660L and diagnoses belonging to ICD chapter XV (Pregnancy, childbirth and the puerperium). The GeneAtlas provides 22 diagnoses classified under ICD chapter XV. Odds ratios (OR) and p-values are computed by GeneAtlas. P-values adjusted for multiple comparison (i.e., controlled for false discovery rate) are given in the rightmost column. P-values <0.05 in bold.
